# Supplementary material for: Regional Variety of Reduction in Retinal Thickness of Diabetic Macular Edema after Anti-VEGF Treatment
Source: Medicina (Kaunas). 2022 Jul 14;58(7):933. doi: 10.3390/medicina58070933 (PMC9321650; doi:10.3390/medicina58070933)
Supplement: Supplementary file 1 [file medicina-58-00933-s001.zip › medicina-1763737-supplementary.pdf]

Table S1. Baseline characteristics at the time of registration

	All patients (n=46)
Age (year)	65.76 ± 12.18
Male gender	35/46 (76.1%)
History of vitrectomy	4/46 (8.7%)
History of cataract surgery	20/46 (43.5%)
History of PRP	26/46 (56.5%)
Creatinine (mg/dl)	0.92 ± 0.32
Hemoglobin A1c (%)	7.60 ± 1.35
Duration of DM (years)	13.11 ± 12.07
Aflibercept used for treatment	42/46 (91.3%)

PRP; pan retinal photocoagulation

DM; diabetes mellitus
